# Estrogen Signaling Influences Nephron Segmentation of the Zebrafish Embryonic Kidney

**DOI:** 10.3390/cells12040666

**Published:** 2023-02-20

**Authors:** Hannah M. Wesselman, Allison E. Gatz, Mairead R. Pfaff, Liana Arceri, Rebecca A. Wingert

**Affiliations:** Department of Biological Sciences, Center for Stem Cells and Regenerative Medicine, Center for Zebrafish Research, Boler-Parseghian Center for Rare and Neglected Diseases, Warren Center for Drug Discovery, University of Notre Dame, Notre Dame, IN 46556, USA

**Keywords:** kidney, nephron, estrogen, xenoestrogen, segmentation, patterning, distal tubule, *esr2b*

## Abstract

Despite significant advances in understanding nephron segment patterning, many questions remain about the underlying genes and signaling pathways that orchestrate renal progenitor cell fate choices and regulate differentiation. In an effort to identify elusive regulators of nephron segmentation, our lab conducted a high-throughput drug screen using a bioactive chemical library and developing zebrafish, which are a conserved vertebrate model and particularly conducive to large-scale screening approaches. 17β-estradiol (E2), which is the dominant form of estrogen in vertebrates, was a particularly interesting hit from this screen. E2 has been extensively studied in the context of gonad development, but roles for E2 in nephron development were unknown. Here, we report that exogenous estrogen treatments affect distal tubule composition, namely, causing an increase in the distal early segment and a decrease in the neighboring distal late. These changes were noted early in development but were not due to changes in cell dynamics. Interestingly, exposure to the xenoestrogens ethinylestradiol and genistein yielded the same changes in distal segments. Further, upon treatment with an estrogen receptor 2 (Esr2) antagonist, PHTPP, we observed the opposite phenotypes. Similarly, genetic deficiency of the Esr2 analog, *esr2b*, revealed phenotypes consistent with that of PHTPP treatment. Inhibition of E2 signaling also resulted in decreased expression of essential distal transcription factors, *irx3b* and its target *irx1a*. These data suggest that estrogenic compounds are essential for distal segment fate during nephrogenesis in the zebrafish pronephros and expand our fundamental understanding of hormone function during kidney organogenesis.

## 1. Introduction

The kidney is a vital organ that facilitates waste excretion, osmoregulation, and fluid homeostasis [1]. Kidney development is a complex process, giving rise to specialized epithelial functional units called nephrons [2,3]. While nephron numbers can greatly vary across species, nephron segmentation and overall function remain highly conserved in vertebrates [4,5]. Namely, the glomerulus functions to filter blood while the subsequent tubular segments modify the filtrate to assure proper secretion and reabsorption, and finally, waste is excreted through the collecting duct [6,7,8,9,10,11,12,13,14,15]. Each of the tubular segments carry out specialized tasks [8,9,10,11,12,13,14,15]. For example, the proximal convoluted tubule is responsible for secreting ammonia, which functions to adjust filtrate pH [8,9,10,11,12,13,14,15]. Later segments, such as the distal convoluted tubule, facilitate sodium and chloride reabsorption [8,9,10,11,12,13,14,15]. While the function and expression signatures of each segment are becoming increasingly better characterized [16], the molecular cues required to guide differentiation of these discrete nephron cell types remain poorly understood.

The zebrafish embryonic kidney, or pronephros, is highly amenable to parsing out these genetic components for several reasons. The architecture of the pronephros is simple, comprised of only two nephrons [17]. The nephrons possess a similar array of functional segments that contain cell types which are analogous to the mammalian nephron [18]. This is consistent with a high degree of genetic conservation between zebrafish and humans [5,19,20]. Coupled with rapid ex utero development, the zebrafish serves as a suitable model for high-throughput genetic and biochemical screens, specifically in the context of renal development to study renal progenitor patterning [21,22,23,24,25,26]. Using the zebrafish, for example, studies have identified novel regulators of nephron segmentation, including the Iroquois transcription factor Irx3b, which is essential for distal early (DE) segment formation [27]. A recent forward genetic screen identified the transcription factor AP-2 alpha (Tfap2a) and its downstream target *irx1a* as key factors in the DE terminal differentiation program [28,29]. Formation of the distal late (DL) segment is known to involve several transcription factors and signaling pathways [30,31,32,33,34,35,36,37]. Even with these advances, there remain significant gaps in our understanding of the mechanisms that drive progenitor cell fate decisions.

In an effort to uncover novel regulators of nephron segmentation, a recent study conducted a high-throughput screen of known bioactive molecules [38,39]. Results from this screen suggested that 17-beta (β) estradiol (or E2, the most dominant form of estrogen in vertebrates) could be a player in nephron development [39]. Here, we follow-up with these results, and report that indeed, estrogen signaling contributes to the processes of nephron segmentation. More specifically, E2 operates throughout development, as early as the 20 somite stage (ss), and contributes to DE/DL segment patterning. From a screen of selective estrogen receptor modulators (SERMs) and genetic studies, we found that E2 specifically operates through *esr2b* to promote DE fate at the expense of the DL. Finally, we found that *esr2b* works upstream of essential transcription factors, *irx3b* and *irx1a*, to confer distal cell fate. Together, these findings implicate estrogen signaling as an essential regulator of nephron segmentation.

## 2. Materials and Methods

### 2.1. Ethics Statement and Zebrafish Husbandry

The Center for Zebrafish Research at the University of Notre Dame maintained the zebrafish used in these studies and experiments were performed with the approval of the University of Notre Dame Institutional Animal Care and Use Committee (IACUC), under protocol numbers 19-06-5412, 20-09-6240, and 22-07-7335.

### 2.2. Animal Models

Tübingen strain wild-type (WT) zebrafish were used for the reported studies unless otherwise noted. The *esr2b* mutant line, Uab127, was graciously provided by Dr. Gorelick’s lab at the University of Alabama at Birmingham [40,41]. Zebrafish were raised and staged as described [42]. For all experiments, embryos were incubated in E3 medium at 28 °C until the desired developmental stage, anesthetized with 0.02% tricaine, and fixed using 4% paraformaldehyde/1x PBS (PFA) [43,44]. Embryos were analyzed before sex determination, so we cannot report the effect of sex and gender in the context of this study. 

### 2.3. Whole Mount and Fluorescent in situ Hybridization (WISH, FISH)

WISH was performed as previously described [45,46,47,48,49] with antisense RNA probes, either digoxigenin-labeled (*cdh17*, *odf3b*, *slc20a1a*, *trpm7*, *slc12a1*, *slc12a3*, *kcnj1a.1*, *tbx2b*, *irx1a*, *irx3b*) or fluorescein-labeled (*smyhc*), using in vitro transcription from IMAGE clone templates, as previously described [18,27]. FISH was performed as described using TSA Plus Fluorescein or Cyanine Kits (Table 1) [45,46,47,48,49]. For gene expression studies, every analysis was performed in triplicate for each genetic model with sample sizes of *n* > 20 per replicate.

### 2.4. Immunofluorescence (IF)

Whole mount IF experiments were completed as previously described on PFA-fixed embryo samples, with primary and secondary antibodies as listed in Table 1 [43,50,51,52]. Proliferating cells were marked with anti-PH3 diluted 1:200 and apoptosis was marked with anti-activated Caspase3 at 1:50.

### 2.5. Chemical Treatments

Chemical treatments were completed as previously described, with chemicals as listed in Table 1 [34,38,39]. Chemicals were dissolved in DMSO to make a 10 mM stock solution. Stocks were aliquoted and stored at −80 °C. Aliquots were thawed at room temperature and protected from light. Working solutions were diluted in E3 and distributed to 6- or 12-well plates. Chemical treatments were completed beginning at the shield stage (6 h post-fertilization (hpf)) until the 24 hpf stage, unless otherwise noted. We chose to treat at the shield stage, as the animals were already undergoing gastrulation. Thus, this timepoint prevents interference with the onset of gastrulation. The dose for each chemical was decided by treating at doses consistent with previous studies or slightly increased concentrations to maximize penetrance while minimizing morphological defects. Animals treated with E2 exhibited distal segmentation phenotypes at both 20 µM and 25 µM. As most animals had a curved body axis at 25 µM, we proceeded with 20 µM treatments. We treated with 400 µM DPN, 400 µM MPP, and 75 µM PPT, however the animals did not exhibit changes in distal nephron segmentation. When exposed to higher doses, the animals exhibited morphological defects or mortality. PHTPP exhibited the highest penetrance of distal segmentation without morphological defects at 18 µM. Xenoestrogens genistein and ethinylestradiol exhibited the highest penetrance of distal segmentation without morphological defects at 20 µM. Treatments were conducted in triplicate with at least *n* > 20 embryos per replicate at various doses (Table 2). All experiments were conducted with a DMSO vehicle control. DMSO control animals are demarcated as “WT” in all graphics and schematics. 

### 2.6. Genetic Models

Antisense morpholino oligonucleotides (MOs) were obtained from Gene Tools, LLC (Philomath, OR, USA). MOs were solubilized in DNase/RNase-free water to generate 4 mM stock solutions, which were stored at 20 °C. Zebrafish embryos were injected at the 1-cell stage with 5 nL of diluted MO. Optimal dosage was determined by previously published doses and our own experience [53]. *esr1* was targeted with 5′–catgtaaaacaggctggtcacCTTG–3′ (0.4 mM). Esr2a was targeted with 5′–agagagtcttacCTTGTATACTC–3′ (0.8 mM). Esr2b was targeted with 5′–ttgaccatgagcattacCTTGAATG–3′ (0.8 mM) [53]. Uab127 embryos were genotyped with the forward primer 5′–GTCCCGCTTAGTCCCACAAT–3′ and the reverse primer 5′–TGACAGCTGCCACCTAAAGA–3′ [54].

### 2.7. Image Acquisition

A Nikon Eclipse Ni with a DS-Fi2 camera was used to image WISH samples and live zebrafish. Live zebrafish were mounted in methylcellulose with trace amounts of tricaine present. IF and FISH images were acquired using a Nikon C2 confocal microscope or a Nikon A1R confocal microscope.

### 2.8. Quantification and Statistical Analysis 

Each experiment was completed in a minimum of triplicates. Analysis of all experimental work was performed in a blinded manner. From these measurements, an average and standard deviation (SD) were calculated, and t-tests or ANOVA tests were completed to compare control and experimental measurements using GraphPad Prism 9 software. Statistical details for each experiment are located in the corresponding figure legend.

## 3. Results

### 3.1. Exogenous E2 Treatment Alters Nephron Segmentation

**Figure 1:**
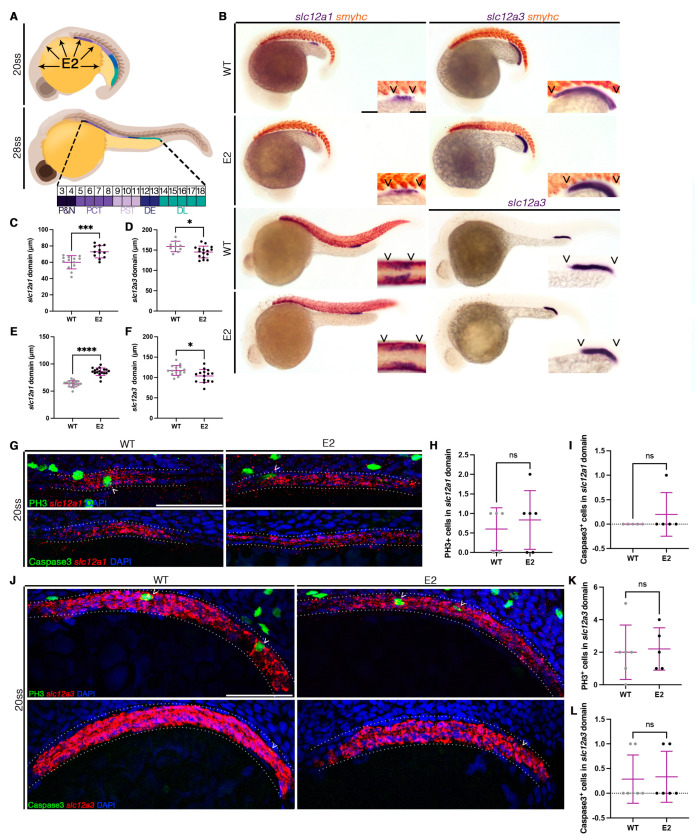
Exogenous E2 alters distal nephron segmentation independently of cell dynamics. (**A**) Schematic of nephrogenesis in a zebrafish from the early 20 ss (top) to the fully patterned 28 ss (bottom). 17β-estradiol (E2) is present in the yolk ball and diffuses to surrounding tissues. By 28 ss, the nephron is comprised of discrete segments with unique somite addresses: podocytes and neck (P&N, somites 3–4), proximal convoluted tubule (PCT, somites 5–8), proximal straight tubule (PST, somites 9–11), distal early (DE, somites 12–13), and distal late (DL, somites 14–18). (**B**) 20 ss (top two rows) and 28 ss (bottom two rows) WT control animals treated with DMSO as a vehicle control, and siblings treated with 20 µM of E2 from the shield stage to 24 hpf, stained via WISH for the DE marker (*slc12a1*, left) or the DL marker (*slc12a3*, right) with the somite marker (*smyhc*). Scale bar = 100 µm for lower magnification, scale bar = 50 µm for higher magnification. (**C**,**D**) DE and DL domain lengths at 20 ss in micrometers. (**E**,**F**) DE and DL domain lengths at 28 ss in micrometers. (**G**) The 20 ss WT (left) and 20 µM E2 (right) nephrons (outlined with dotted line) stained for the DE (*slc12a1*) via FISH and proliferating (PH3, top) or apoptotic (Caspase3, bottom) cells via immunofluorescence. Arrow heads denote double-positive cells. Scale bar = 50 µm. (**H**) Number of PH3-positive cells in the DE at the 20 ss. (**I**) Number of Caspase3-positive cells in the DE at the 20 ss. (**J**) The 20 ss WT (left) and 20 µM E2 (right) nephrons (outlined with dotted lines) stained for the DL (*slc12a3*) via FISH and proliferating (PH3, top) or apoptotic (Caspase3, bottom) cells via immunofluorescence. Arrow heads denote double-positive cells. Scale bar = 50 µm. (**K**) Number of PH3-positive cells in the DL at the 20 ss. (**L**) Number of Caspase3-positive cells in the DL at the 20 ss. Data presented on graphs are represented as mean ± SD. * *p* < 0.05, *** *p* < 0.001, and **** *p* < 0.0001 (*t*-test).

The zebrafish has long been established as a valuable high-throughput model to interrogate various biological processes using small molecules [38,55,56,57]. The zebrafish is particularly amenable to studying kidney development, as the functional units, called nephrons, are comprised of highly conserved segments that are patterned by 24 h post-fertilization (hpf). In a chemical screen using known bioactives, researchers identified potential regulators contributing to nephron segmentation [39]. Among the screen hits, 17-beta estradiol (E2, the most dominant form of estrogen in vertebrates) treatment resulted in changes in segmentation [39]. This was particularly interesting, as previous work has established that estrogen response elements (ERE) are active in the pronephros at 18 h post-fertilization (hpf) [58]. Furthermore, estrogen is present in the yolk, at a rate almost 7 times higher than the rest of the animal [58]. We therefore hypothesized that this potent chemical diffuses into the adjacent intermediate mesoderm, thereby influencing pronephric development, specifically segmentation of the nephron by 24 hpf (Figure 1A). To test this, we examined if nephron segmentation was influenced by exogenous E2. We treated embryos at different doses of E2 beginning at the shield stage, then allowed animals to develop to the stage of interest. At an E2 dosage of 20 µM, animals exhibited changes in the distal nephron as early as the 20 ss (Figure 1B). Specifically, the DE segment expanded (Figure 1C) while the DL was shortened (Figure 1D). This phenotype persisted to the 28 ss when the nephron segments were initially patterned (Figure 1B,E,F).

To confirm that these changes were a true change in cell identity rather than changes in *slc12a1* or *slc12a3* independently, we used additional markers for the DE and DL (*kcnj1a.1* and *tbx2b*, respectively). Indeed, we found that the observed DE and DL domain changes were recapitulated with these alternative cell type markers (Figure S1). Additionally, treatment of E2 did not result in changes in the PCT (*slc20a1a*) or PST (*trpm7*) (Figure S1). Additionally, the time of addition did not appear to affect the penetrance of the observed phenotypes (Figure S2). However, we chose to continue with treatment at 6 hpf for subsequent treatments, as this time is most conducive to a high-throughput workflow. We also treated animals at increased concentrations, including 25 µM. Overall, the increased concentration phenocopied those observe at 20 µM, including increased DE and decreased DL (Figure S3). Animals treated at 25 µM did not exhibit changes in other cell types or the overall nephron length, suggesting that the observed phenotypes were segment-specific rather than affecting the formation of the entire tubule (Figure S3). However, the 25 µM treatment did result in greater morphological differences, such as body curvature (Figure S3). Therefore, we continued with treatments at 20 µM as we observed the highest penetrance of phenotypes, while still producing animals with overall morphologically normal-appearing body plans. 

As the observed changes in DE and DL formation may be caused by alterations in cell dynamics or changes in cell fate decisions, we next investigated cell death and proliferation in the segments of interest. We chose to evaluate these characteristics at the 20 ss, the earliest time point at which we observed altered segmentation. Interestingly, there was no significant difference in proliferating cells, as marked by Phospho-Histone H3 (PH3) in the DE segment (Figure 1G,H). Additionally, no changes in cell death as marked by activated Caspase3 were detected (Figure 1G,I). Similarly, there was no significant difference in proliferating cells nor cell death in the DL segment (Figure 1J–L). These data suggest that exogenous E2 treatment does not affect cell dynamics, but rather, influences nephron patterning, specifically of the DE and DL.

### 3.2. Xenoestrogen Treatment Recapitulates E2 Pronephros Segment Phenotypes 

**Figure 2:**
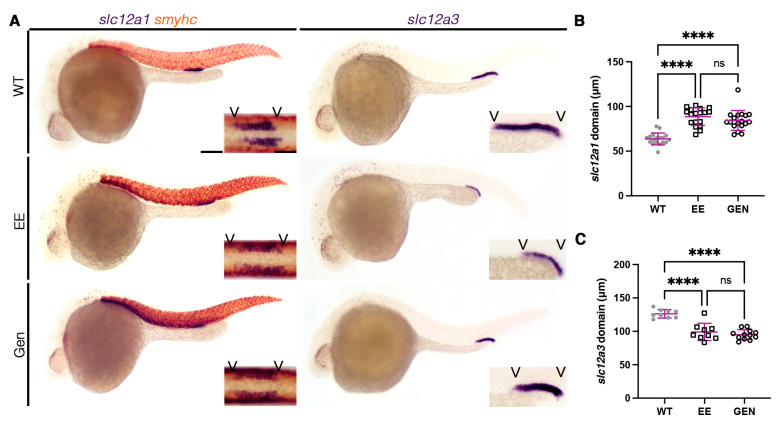
Xenoestrogens phenocopy E2 distal nephron segmentation phenotypes. (**A**) The 28 ss WT (top), 20 µM ethinylestradiol (EE, middle), or 20 µM genistein (GEN, bottom) treated animals stained via WISH for the DE marker (*slc12a1*) with the somite marker (*smyhc*) (left), or the DL marker (*slc12a3*) (right). Scale bar = 100 µm for lower magnification, scale bar = 50 µm for higher magnification. (**B**,**C**) DE and DL domain lengths at 28 ss in micrometers. Data presented on graphs are represented as mean ± SD. **** *p* < 0.0001 (ANOVA).

Xenoestrogens are potent teratogens that have been previously shown to activate E2 signaling pathways similarly to E2 [58,59]. We treated with three compounds: genistein, found in soy products, ethinylestradiol, commonly used in contraceptives, and bisphenol A (BPA), a hardening agent in plastic. As seen with E2 treatment, 20 µM of ethinylestradiol and genistein resulted in an increase in the DE domain (Figure 2A,B). These two xenoestrogens also caused a coordinated decrease in the DL domain (Figure 2A,C). Similar to that of E2, treatment of ethinylestradiol and genistein also altered the expression of other DE and DL markers (Figure S4C–E), while the more proximal segments remained unchanged (Figure S4F–H). Unlike ethinylestradiol and genistein, BPA did not affect the DE domain length (Figure S4A,B).

To assure the drug was not degraded, we evaluated other previously identified phenotypes induced by BPA, such as otolith malformations [60]. Indeed, 50 µM treatment of BPA induced altered otolith formation, suggesting that while the chemical was active, it does not phenocopy E2 in the kidney (Figure S4). BPA has been previously shown to bind preferentially to Esr1 in the zebrafish and exhibits less potent phenotypes in other tissues, suggesting that the lack of a distal phenotype may be due to chemical–receptor interactions [58,59]. Nonetheless, estrogen signaling, regardless of its activating agent (E2, ethinylestradiol, or genistein), appears to influence distal nephron segmentation.

### 3.3. E2 Acts through Esr2b to Confer Distal Segment Changes in the Nephron

**Figure 3:**
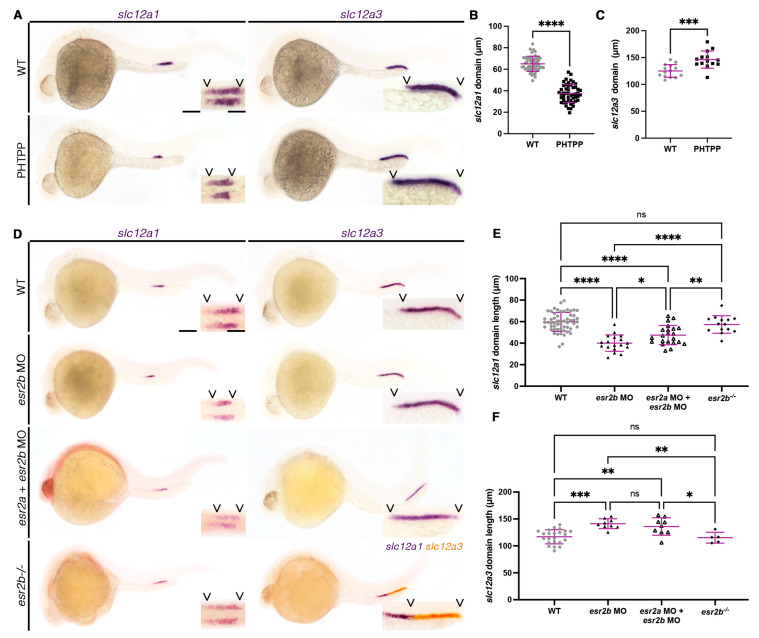
Chemical and genetic inhibition of Esr2b results in altered distal segmentation. (**A**) The 28 ss WT (top) and 18 µM PHTPP (bottom) treated animals stained via WISH for the DE marker (*slc12a1*) (left) or the DL marker (*slc12a3*) (right). Scale bar = 100 µm for lower magnification, scale bar = 50 µm for higher magnification. (**B**,**C**) DE and DL domain lengths at 28 ss in micrometers. (**D**) The 28 ss WT (top), *esr2b* MO-injected (second row), *esr2a* and *esr2b* double-MO-injected (third row), and *esr2b* mutant (bottom row) animals stained via WISH for the DE marker (*slc12a1*) (left) or the DL marker (*slc12a3*) (right). Scale bar = 100 µm for lower magnification, scale bar = 50 µm for higher magnification. (**E**,**F**) DE and DL domain lengths at 28 ss in micrometers. Data presented on graphs are represented as mean ± SD. * *p* < 0.05, ** *p* < 0.01, *** *p* < 0.001, and **** *p* < 0.0001 (*t*-test or ANOVA).

As ethinylestradiol, genistein, and E2 appeared to alter the distal cell fate, we next interrogated the mechanism by which this occurs. Since EREs are active in the nephron early in development, we specifically explored estrogen receptors that operate as ligand-activated transcription factors. In the zebrafish, this includes Esr1, Esr2a, and Esr2b. We first used a targeted chemical screen of selective estrogen receptor modulators (SERMs), including MPP (Esr1 antagonist), PPT (Esr1 agonist), PHTPP (Esr2 antagonist), and DPN (Esr2 agonist). From this screen, an 18 µM treatment of PHTPP, a pan-antagonist of Esr2a and Esr2b, resulted in a decrease of the DE domain and an increase in the DL (Figure 3A–C). Interestingly, DPN, the putative pan-agonist for Esr2a and Esr2b, did not result in an increase in the DE domain (Figure S5). While DPN has been shown to clearly antagonize human ESR2, recent studies have revealed that subtle differences exist between the activation of ESR2 in humans and Esr2a and Esr2b in zebrafish, specifically regarding activation by SERMs [59]. Interestingly, DPN has been shown to preferentially activate Esr1 over Esr2a or Esr2b in zebrafish, which may have prevented an observable phenotype in the DE [59]. Similarly, neither MPP nor PPT resulted in altered distal segments (Figure S5A,B). We further confirmed that PHTPP was specifically affecting the distal segments by measuring the *kcnj1a.1* and *tbx2b* domains, alternate markers for the DE and DL, respectively. Similar to *slc12a1*, the *kcnj1a.1* domain was significantly decreased while the *tbx2b* domain recapitulated the *slc12a3* domain, which was significantly increased (Figure S6). Additionally, the proximal segments (PCT and PST) remained unchanged (Figure S6). Together, the results from the SERM screen suggest that estrogen signaling may be acting via *esr2a* or *esr2b* in nephron segmentation. 

To confirm the results of the SERM screen and further understand which receptor may be the primary player in the kidney, we used morpholinos targeting *esr1*, *esr2a*, and *esr2b* to knockdown each receptor, respectively. Each of these tools was previously validated to interfere with splicing for each specific target, while off-target alterations in splicing were not observed [53]. Knockdown of *esr1* and *esr2a* did not result in changes to the DE (Figure S7). However, *esr2b* deficiency resulted in a decreased DE, and increased the length of the DL, which recapitulates the PHTPP phenotype, though this occurred at a decreased penetrance compared to PHTPP treatment (Figure 3D–F). We hypothesized that *esr2b* may be the major player in distal cell development. However, it is plausible that *esr2a* may serve a redundant role. To address this, we knocked down *esr2a* and *esr2b* in combination. The double-deficient animals did not exhibit more severe differences in DE or DL segmentation compared to the single *esr2b*-deficient animals (Figure 3D–F). This led us to hypothesize that Esr2a and Esr2b likely do not function redundantly in the context of conferring nephron cell fate. 

While morpholinos offer valuable insight to genetic mechanisms in development, we were also interested in exploring the role of *esr2b* in the context of a stable genetic mutant. We obtained the *esr2b^uab127^* line, which contains a 5 base pair deletion resulting in a premature stop codon before the DNA-binding domain [40,41]. Interestingly, *esr2b*^−/−^ animals did not exhibit significant changes in the DE or DL domains (Figure 3D–F). From pairwise matings of *esr2b*^+/−^ parents, none of the progeny, including heterozygotes, were significantly different from one another in the DE nor the DL (Figure 3, Figure S7). However, we hypothesize that this is due to mature and robust maternally deposited transcripts encoding Esr2b, which have been previously detected [58]. Due to a previously reported fertility defect in homozygous *esr2b*^−/−^ females, we could not evaluate maternal zygotic mutant embryos [40]. However, the consistency between PHTPP treatment and *esr2b* MO is consistent with our hypothesis that estrogen signaling influences distal segmentation. 

### 3.4. Alterations in Estrogen Signaling Associated with Changes in Expression of Distal Segment Transcription Factors Irx3b and Irx1a in the Pronephros

**Figure 4:**
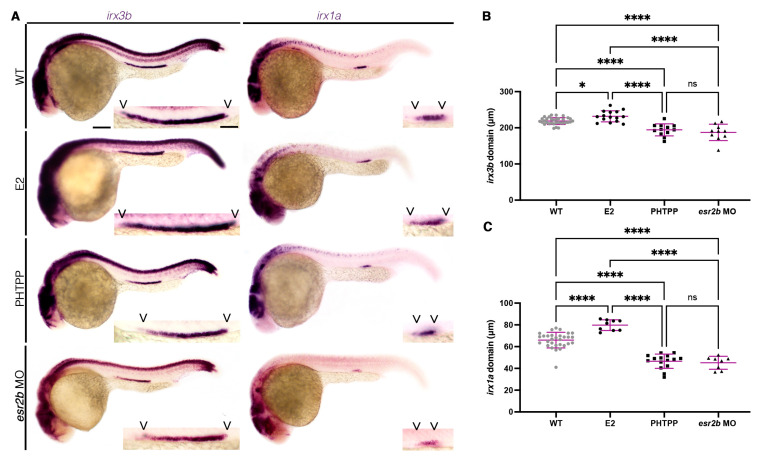
E2 signaling operates through Esr2b upstream of essential transcription factors to elicit distal nephron cell fate. (**A**) The 28 ss WT (top), 20 µM E2-treated (second row), 18 µM PHTPP-treated (third row), or *esr2b* MO-injected (bottom) animals stained via WISH for the transcription factor *irx3b* (left) and its downstream target *irx1a* (right). Scale bar = 100 µm for lower magnification, scale bar = 50 µm for higher magnification. (**B**,**C**) The *irx3b* and *irx1a* domain lengths at 28 ss in micrometers. Data presented on graphs are represented as mean ± SD. * *p* < 0.05 and **** *p* < 0.0001 (ANOVA).

We next sought to explore the mechanism by which E2 and *esr2b* act in zebrafish embryonic nephrogenesis. Previous work has established several roles for the transcription factor Irx3b and its downstream target, Irx1a, in nephron segmentation [27,28,29,61,62,63,64,65,66]. Specifically, in the zebrafish, Irx3b is essential for adoption of the DE lineage identity, where downstream expression of Irx1a is sufficient to support adoption of this identity [27,28]. Using WISH, we interrogated the effect of E2 and PHTPP on *irx3b* expression, respectively. Interestingly, we found that E2 resulted in an expansion of the *irx3b* pronephros domain at both 20 µM and 25 µM doses (Figure 4A–C, Figure S3). Conversely, PHTPP exhibited the opposite phenotype with a truncation of the *irx3b* domain (Figure 4A–C, Figure S3). Similarly, E2 treatment expanded the *irx1a* pronephros domain, while PHTPP decreased *irx1a* expression. Together, these data suggest that E2 signaling acts through Esr2b, upstream of Irx3b and Irx1a, to determine distal nephron segmentation.

## 4. Discussion

While traditionally associated with women’s health, estrogen signaling has long been established as a regulator of developmental processes. Outside of gonad development, estrogen signaling also influences the development of the brain [67,68,69,70,71,72], hematopoietic stem cell niche [58,73], kidney [74,75], prostate and lung [76], among others [77]. The primary ligand of estrogen signaling in vertebrates is 17β-estradiol (E2), which can bind three receptors in mammals (ESR1 (ERa), ESR2 (ERb), and GPER) and four receptors in zebrafish (Esr1, Esr2a, Esr2b, and Gper). Notably, the highly conserved functional domains (DBD and LBD) have sequence homology upwards of 70% between zebrafish and humans [75]. Furthermore, zebrafish receptors are activated in a similar manner to their human counterparts by the same ligands [59]. For these reasons, along with their powerful genetics and tractability for developmental studies, zebrafish have been a useful model for expanding our understanding of estrogen signaling in ontogeny. The results from a recent bioactive screen completed by our lab were of particular interest, as they suggested for the first time that estrogen may play an explicit role in the process of nephron segmentation [39]. These initial observations were only superficial, however, and required additional follow-up studies in order to elucidate the molecular details.

Here, we have used the zebrafish as a model for nephrogenesis to interrogate the role of estrogen signaling, with a focus on E2 and early segmentation during the formation of the first kidney, or embryonic pronephros. We found that exogenous E2 alters DE and DL segment domains without changing cell death or proliferation in those areas, suggesting that estrogen signaling contributes to the patterning of renal progenitors that generate these populations. This is rather surprising, as estrogen and related derivatives have been shown to inhibit proliferation in the brain [78]. In contrast, our findings suggested that E2 signaling may be controlling cell fate decisions, which has also been observed in other tissues [54]. For example, in the liver, hepatocytes and biliary epithelial cells arise from a common hepatoblast progenitor, and researchers found that E2 acting specifically through the receptor Esr2b promoted commitment to the hepatocyte fate both in zebrafish and human hepatoblast culture [54]. Previous studies have also demonstrated that estrogen regulates key transporter expression and function (Aquaporin2) in the kidney, which is consistent with our observations in altered transporters *slc12a1* and *slc12a3* expression [79]. Though, it remains unknown if these changes in transporter expression have an effect on kidney physiology. 

Additionally, consistent with prior work, we found that xenoestrogens ethiniylestradiol and genistein act similarly to E2 [58,59]. Xenoestrogens, or ‘foreign’ estrogens, are widely found throughout our environment and in man-made products such as plastics. Similar enough in structure to mimic naturally occurring estrogen, they can bind to estrogen receptors and induce potentially harmful outcomes, such as birth defects. Thus, elucidating the effects of these compounds is relevant to understanding environmental factors of conditions such as congenital anomalies of the kidney and urinary tract (CAKUT) [80,81]. Our work emphasized the ability of xenoestrogens to elicit potent effects on developing tissues.

**Figure 5:**
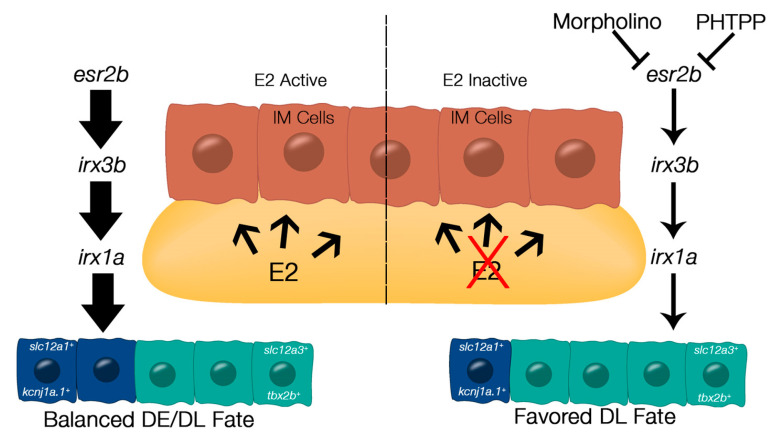
Proposed mechanism by which E2 signaling contributes to nephron segmentation. When E2 signaling is active (left), E2 diffuses from the yolk into the neighboring intermediate mesoderm (IM) cells. E2 binds to Esr2b, which activates *irx3b* and *irx1a*, ultimately resulting in balanced DE and DL fate. When E2 signaling is inactive (right) via morpholino knockdown or chemical inhibition by PHTPP, *esr2b* activity is diminished, as well as expression of downstream targets *irx3b* and *irx1a*, resulting in favored DL fate.

Next, our targeted SERM screen further suggested that either Esr2a or Esr2b was responsible for the segmentation changes, as PHTPP resulted in a decreased DE and increased DL. Only knockdown of *esr2b* recapitulated the PHTPP phenotype, leading us to hypothesize that Esr2b is the major player for estrogen signaling in the embryonic kidney. Considering that the Irx family of transcription factors, specifically *irx3b* and its downstream target *irx1a*, have been shown to regulate DE cell fate, we next investigated if exogenous E2 or inhibition of Esr2 signaling affected their relative expression domains. Indeed, E2 expanded both *irx3b* and *irx1a* domains, while PHTPP treatment resulted in a truncation. Therefore, we hypothesize that E2 signaling works upstream of these factors (Figure 5). 

Interestingly, we did not observe a segmentation phenotype in *esr2b*^−/−^ animals. This is likely due to maternally deposited transcripts in the early embryo. Previous work has established that both *esr2a* and *esr2b* expression drastically decrease shortly after fertilization, which is consistent with maternal deposition [58]. Due to the infertility of *esr2b*^−/−^ females, we are unable to test this with a stable genetic line. Further, the normal distal segments of *esr2b*^−/−^ animals may be, in part, due to genetic compensation from other estrogen receptors, a common occurrence in stable genetic mutants [82]. Considering our findings, future directions may be interested in the exact mechanism of E2 segmentation for regulation. Here, we suggest candidates *irx3b* and *irx1a*, but it is unknown if Esr2b directly binds the promoter of either of these factors or perhaps as a coactivator for their respective downstream targets. For this reason, co-immunoprecipitation of Esr2b followed by sequencing may be of particular interest. 

Additionally, we chose to focus on nephron segmentation specifically, considering how little is known about the role of estrogen signaling in this process. There have been exciting, continued advances in our understanding of nephron segment patterning in recent years [83,84]. The field has made important discoveries about the landscape of kidney cell types, their expression profiles, and changes in various disease states [16]. Nevertheless, many knowledge gaps remain. For example, the complex interplays between critical genes are not fully understood. The present study illuminates a role for estrogen signaling, but much more research is needed in order to delineate how the activities of this pathway interrelate to the other genetic mechanisms that influence the processes of nephron segmentation.

Future studies may also be interested in how continued E2 treatment through early larval stages affects mesonephric branching. Long-term exposure of E2 and similar estrogenic compounds has been noted to cause morphological defects in zebrafish larva, as we observed in our studies as well, so it is likely that animals would display kidney defects in this experiment [85]. Other studies, however, treated at lower concentrations of estrogen modulators for longer periods of time and have avoided gross morphological deficiencies [75,86]. Interestingly, when embryos are exposed to low concentrations of E2 followed by microarray analysis, researchers found transcriptomic changes in the renal tissue at 3 dpf [75]. The upregulated genes found in this study span various functions, including solute transport, ATP binding, and kinase activity, and many localize to the DE region. While additional studies are required to parse out if these transcriptional changes identified in whole-body lysates confer changes to cellular identity in the kidney, these results still point to the ability of estrogen signaling to modulate pronephric expression profiles. Furthermore, studies regarding the effects of acute versus chronic estrogen exposure may elucidate the nature of these transcriptional changes.

## 5. Conclusions

Embryonic E2 exposure is highly regulated during gestation, though additional estrogenic compounds may be present depending on the environment. In particular, models of oral contraception have been shown to disrupt fetal development [87]. However, the effects of early estrogen exposure may span beyond early development. For example, even transient exposure of estrogenic compounds can alter osteoclast formation into adulthood [88]. Long-term effects may be due to alterations in epigenetics, as gestational administration of E2 can affect DNA methylation even into adulthood [89]. In addition to the long-lasting phenotypes resulting from E2 exposure in other tissues, estrogenic compounds have also been noted as potential therapeutics in various kidney disorders. In proliferative kidney diseases of fish, ethinyl estradiol exposure attenuated disease progression [90]. Estrogen has also mitigated kidney ischemia and reperfusion injury by activating metabolic pathways through PPARγ [91,92]. Estrogen-based hormone replacement in postmenopausal patients also appears to ameliorate kidney dysfunction and slow the progression of chronic kidney disease symptoms [93,94]. Interestingly, estrogen inhibition with tamoxifen also had beneficial effects, as treatment reduced renal fibrosis in human and rat kidneys [95]. Our current study further underscores the need to understand the mechanism by which estrogenic compounds activate these changes in renal tissue. Estrogen, in its various forms, may be reno-protective or damaging, but the fundamental observations reported here are essential in taking a step towards elucidating the role of hormonal signaling in the kidney.

## Figures and Tables

**Table 1:** Reagents and resources used in this study.

Reagent or Resource	Name	Source	Catalog Number
**Antibodies**	Anti-Ph3	Millipore	06–570
	Anti-Caspase3	BD Biosciences	559565
	Goat anti-Mouse, Alexa Fluor 568	Invitrogen	A11031
	Goat anti-Rabbit, Alexa Fluor 594	Invitrogen	A11037
	Goat anti-Mouse, Alexa Fluor 488	Invitrogen	A11029
	Goat anti-Rabbit, Alexa Fluor 488	Invitrogen	A11034
**Chemicals and Stains**	17β-Estradiol (E2)	Cayman Chemical	50-28-2
	DPN	Santa Cruz	SC-203431
	PHTPP	Santa Cruz	SC-204191
	MPP	Santa Cruz	SC-204098
	PPT	Santa Cruz	SC-297946
	Genistein	Sigma Aldrich	446-72-0
	Ethinylestradiol	Sigma Aldrich	47-63-6
	BPA DAPI	Sigma Aldrich Invitrogen	80-05-7 D1306
**Commercial Assays**	mMESSAGE mMACHINE SP6 kit	Ambion	AM1340
	TSA Plus Cyanine	Akoya Biosciences	NEL744001KT
	TSA Plus Fluorescein	Akoya Biosciences	NEL741001KT
**Software**	https://www.graphpad.com, URL accessed on 15 April 2019	Prism v9	GraphPad
	https://imagej.nih.gov/ij/, URL accessed on 2 August 2020	ImageJ	Fiji

**Table 2:** Zebrafish embryo treatment dosages for chemical studies.

Chemical	Dose
17β-Estradiol (E2)	20 µM or 25 µM
DPN	400 µM
PHTPP	18 µM
MPP	400 µM
PPT	75 µM
Genistein	20 µM
Ethinylestradiol	20 µM
BPA	50 µM

## Data Availability

All data are provided herein or within supplementary files.

## Supplementary Materials

The following supporting information can be downloaded at: https://www.mdpi.com/article/10.3390/cells12040666/s1, Figure S1: Further characterization of nephron segment development in E2-treated zebrafish embryos. Figure S2: Assessment of DE segment development following E2 treatment with different times of addition in zebrafish embryos. Figure S3: Characterization of pronephros development following exposure to 25 µM E2. Figure S4: Further characterization of nephron segment development in xenoestrogen-treated zebrafish embryos. Figure S5: Selective estrogen receptor modulators MPP, PPT, and DPN have no effect on DE segment development in the zebrafish pronephros. Figure S6: Effects of Esr2 antagonist PHTPP on nephron segmentation. Figure S7: Evaluation of DE segment development following *esr1* and *esr2* knockdown.
