# Supplementary material for: Estrogen Signaling Influences Nephron Segmentation of the Zebrafish Embryonic Kidney
Source: Cells. 2023 Feb 20;12(4):666. doi: 10.3390/cells12040666 (PMC9955091; doi:10.3390/cells12040666)
Supplement: Supplementary file 1 [file cells-12-00666-s001.zip › cells-2210566-supplementary.pdf]

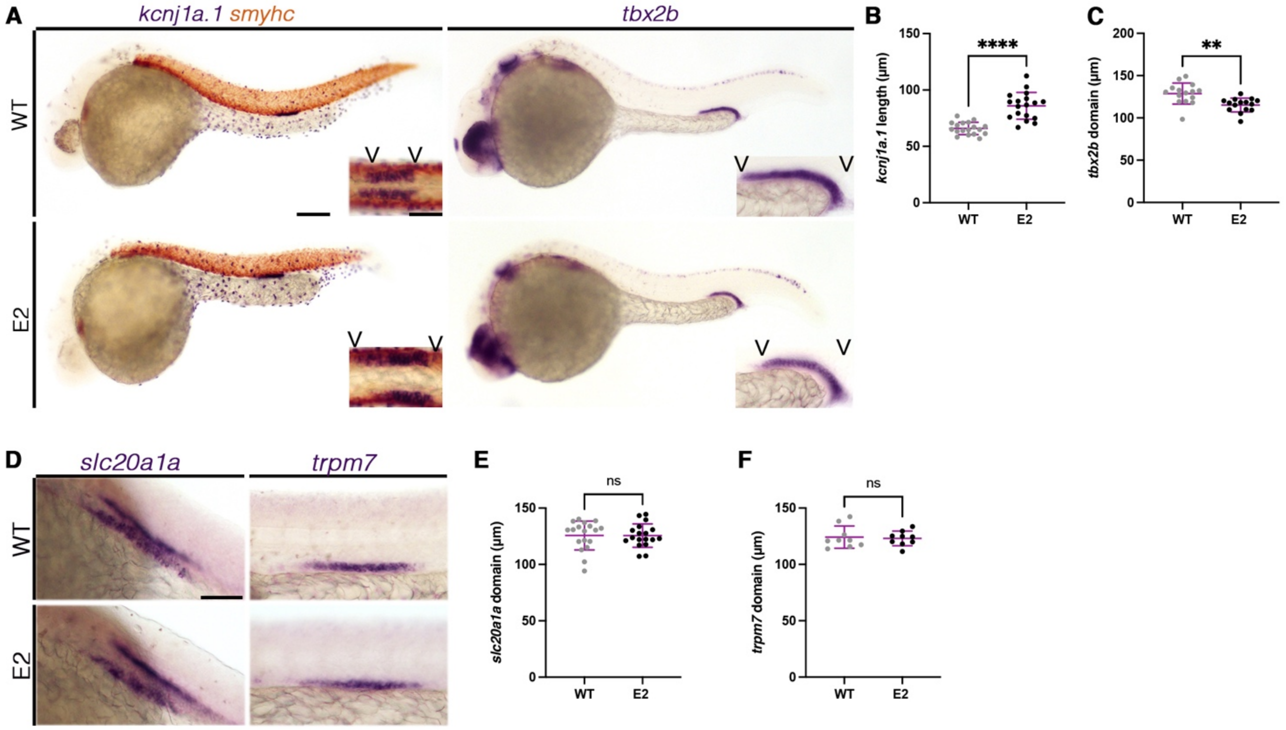

Supplemental Figure S1. Further characterization of nephron segment development in E2 treated zebrafish embryos. (**A**) 28 ss WT (top) 20 μM E2 treated animals (bottom) stained via WISH for the DE marker (*kcnj1a.1*) with somite marker (*smyhc*) (left), or DL marker (*tbx2b*) (right) Scale bar = 100 μm for lower magnification, scale bar = 50 μm for higher magnification. (**B,C**) DE, DL domain length at 28 ss in micrometers**.** (**D**) 28 ss WT (top) 20 μM E2 treated animals (bottom) stained via WISH for the PCT marker (*slc20a1a*) (left) or PST marker (*trpm7*) (right). Scale bar = 50 μm. (**E,F**) PCT, PST domain length at 28 ss in micrometers**.** Data presented on graphs are represented as mean ± SD; **p< 0.01 and ****p < 0.0001 (t test).

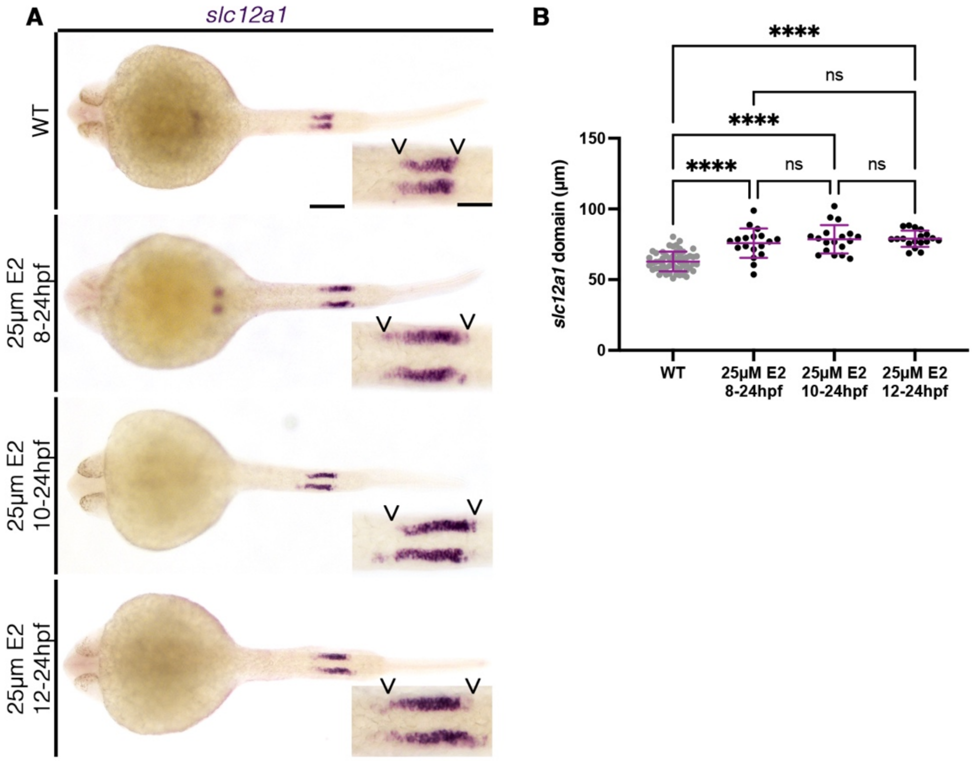

Supplemental Figure S2. DE segment development following E2 treatment with different times of addition in zebrafish embryos. (**A**) 28 ss WT (top) 25 μM E2 treated animals (bottom 3 rows) stained via WISH for the DE marker (*slc12a1*). 25 μM E2 was added at various times (8 hpf, 10 hpf, or 12 hpf) to determine if E2 exhibited reduction in potency over time. Scale bar = 100 μm for lower magnification, scale bar = 50 μm for higher magnification. (**B**) DE domain length at 28 ss in micrometers. Data presented on graphs are represented as mean ± SD; ****p < 0.0001 (ANOVA).

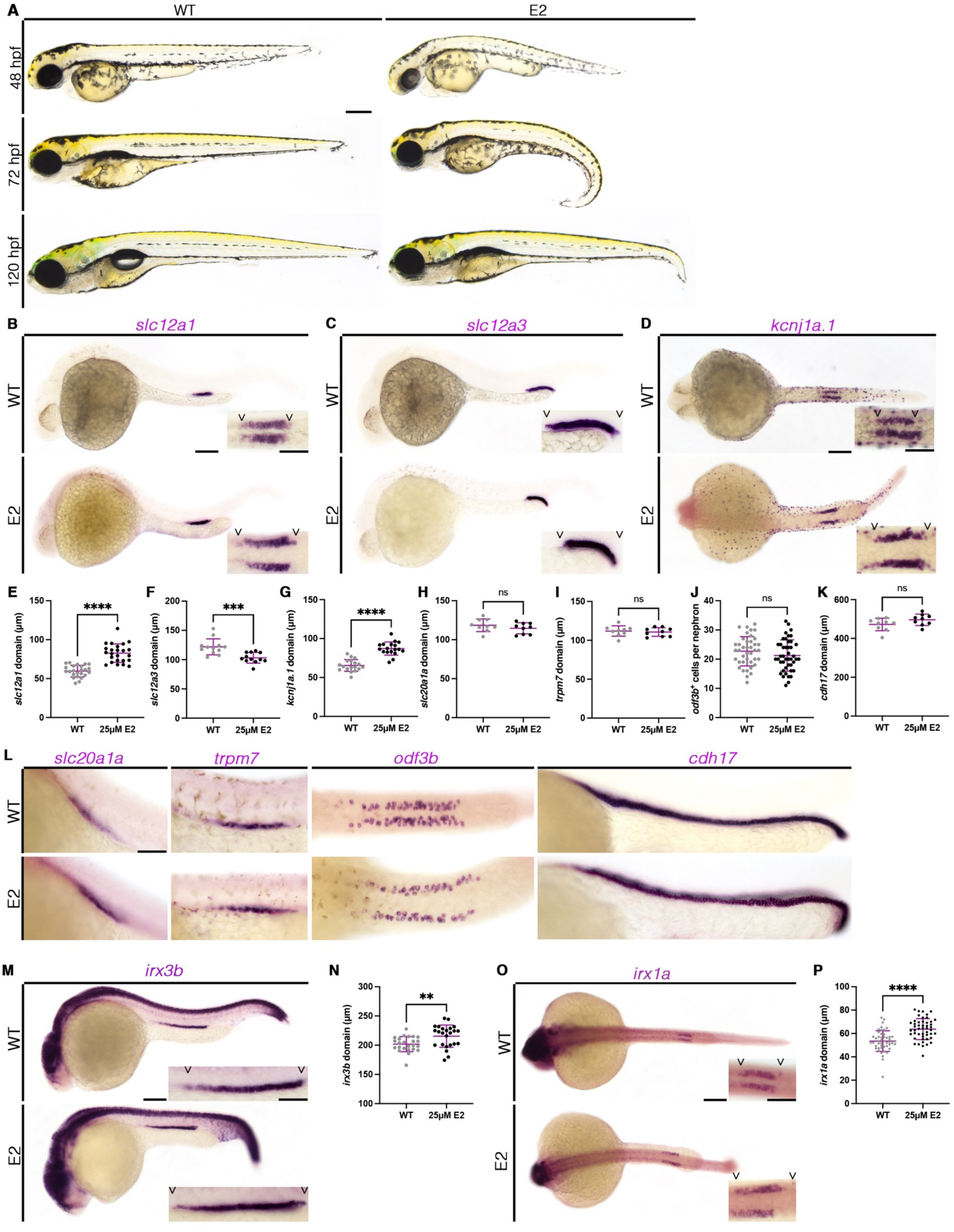
A
WT
E2
48 hpf
72 hpf
120 hpf
B
slc12a1
C
slc12a3
D
kcnj1a.1
WT
E2
E
slc12a1 domain (µm)
****
WT
25µM E2
F
slc12a3 domain (µm)
***
G
kcnj1a.1 domain (µm)
****
H
slc20a1a domain (µm)
ns
I
trpm7 domain (µm)
ns
J
odf3b+ cells per nephron
ns
K
cdh17 domain (µm)
ns
L
slc20a1a
trpm7
odf3b
cdh17
M
irx3b
N
irx3b domain (µm)
**
O
irx1a
P
irx1a domain (µm)
****

Supplemental Figure S3. Assessment of pronephros development following exposure to 25 μM E2. (**A**) Live imaging of imaging of WT (left) and E2 treated animals (right) at various stages. 25 μM E2 or DMSO vehicle control was applied to animals at shield stage and removed at 24 hpf. Animals were washed with E3 and imaged at 48 hpf (top), 72 hpf (middle), and 120 hpf (bottom). Scale bar = 200 μm**.** (**B**) 28 ss WT (top) 25 μM E2 treated animals (bottom) stained via WISH for the DE marker (*slc12a1*) Scale bar = 100 μm for lower magnification, scale bar = 50 μm for higher magnification. (**C**) 28 ss WT (top) 25 μM E2 treated animals (bottom) stained via WISH for the DL marker (*slc12a3*) Scale bar = 100 μm for lower magnification, scale bar = 50 μm for higher magnification. (**D**) 28 ss WT (top) 25 μM E2 treated animals (bottom) stained via WISH for the DE marker (*kcnj1a.1*) Scale bar = 100μm for lower magnification, scale bar = 50 μm for higher magnification. (**E-I**) DE, DL, DE, PCT, PST domain length at 28 ss in micrometers. (**J**) Absolute number of MCCs per nephron. (**K**) Length of the entire nephron tubule at 28 ss in micrometers**.** (**L**) 28 ss WT (top) 25 μM E2 treated animals (bottom) stained via WISH for the PCT (*slc20a1a*), PST (*trpm7*), MCC number (*odf3b*), and total nephron tubule length (*cdh17*). Scale bar = 50 μm. (**M**) 28 ss WT (top) 25 μM E2 treated animals (bottom) stained via WISH for the transcription factor *irx3b* domain. Scale bar = 100 μm for lower magnification, scale bar = 50 μm for higher magnification. (**N**) *irx3b* domain length at 28 ss in micrometers**.** (**O**) 28 ss WT (top) 25μM E2 treated animals (bottom) stained via WISH for the transcription factor *irx1a* domain. Scale bar = 100 μm for lower magnification, scale bar = 50 μm for higher magnification. (**P**) *irx1a* domain length at 28 ss in micrometers**.** Data presented on graphs are represented as mean ± SD; ** $p< 0.01$ ***$p < 0.001$ and ****$p < 0.0001$ (t test).

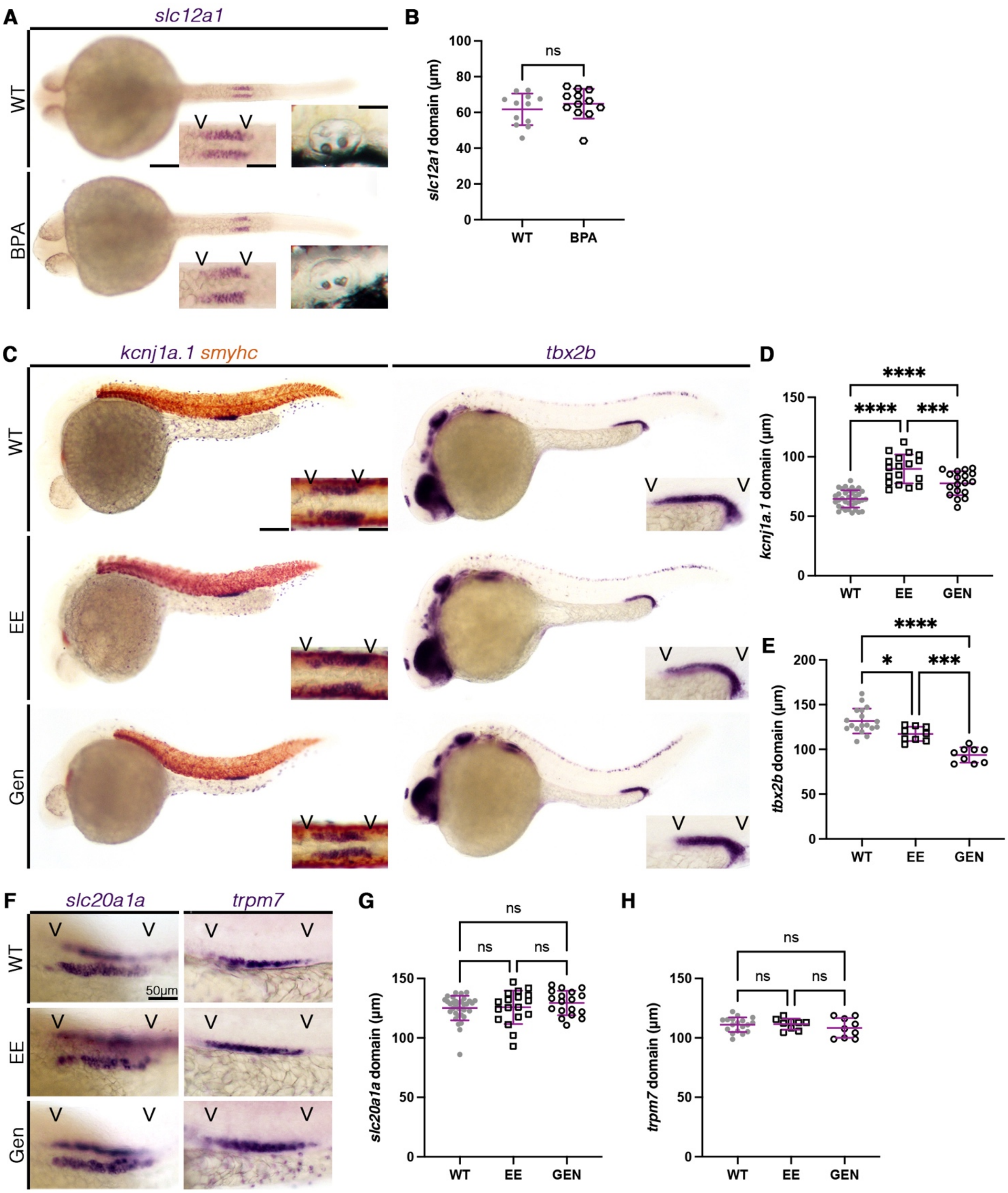

Supplemental Figure S4. Further characterization of nephron segment development in xenoestrogen treated zebrafish embryos. (**A**) 28 ss WT (top) 50 µM BPA treated animals (bottom) stained via WISH for the DE marker (*slc12a1*). Inset is a magnified image of the otolith structure for either WT (top) or 50 µM BPA treated animal (bottom) at 48 hpf. Scale bar = 100 µm for lower magnification, scale bar = 50 µm for higher magnification. (**B**) DE domain length at 28 ss in micrometers**.** (**C**) 28 ss WT (top), 20µM ethinylestradiol (EE, middle), or 20 µM genistein (GEN, bottom) treated animals stained via WISH for the DE marker (*kcnj1a.1*) with somite marker (*smyhc*) (left), or DL marker (*tbx2b*) (right) Scale bar = 100 µm for lower magnification, scale bar = 50 µm for higher magnification. (**D,E**) DE, DL domain length at 28 ss in micrometers. (**F**) 28 ss WT (top), 20 µM ethinyl estradiol (EE, middle), or 20 µM genistein (GEN, bottom) treated animals stained via WISH for the PCT marker (*slc20a1a*) (left) or PST marker (*trpm7*) (right). Scale bar = 50 µm. (**G,H**) PCT, PST domain length at 28 ss in micrometers. Data presented on graphs are represented as mean ± SD; *p<0.05, ***p < 0.001 and ****p < 0.0001 (t test or ANOVA).

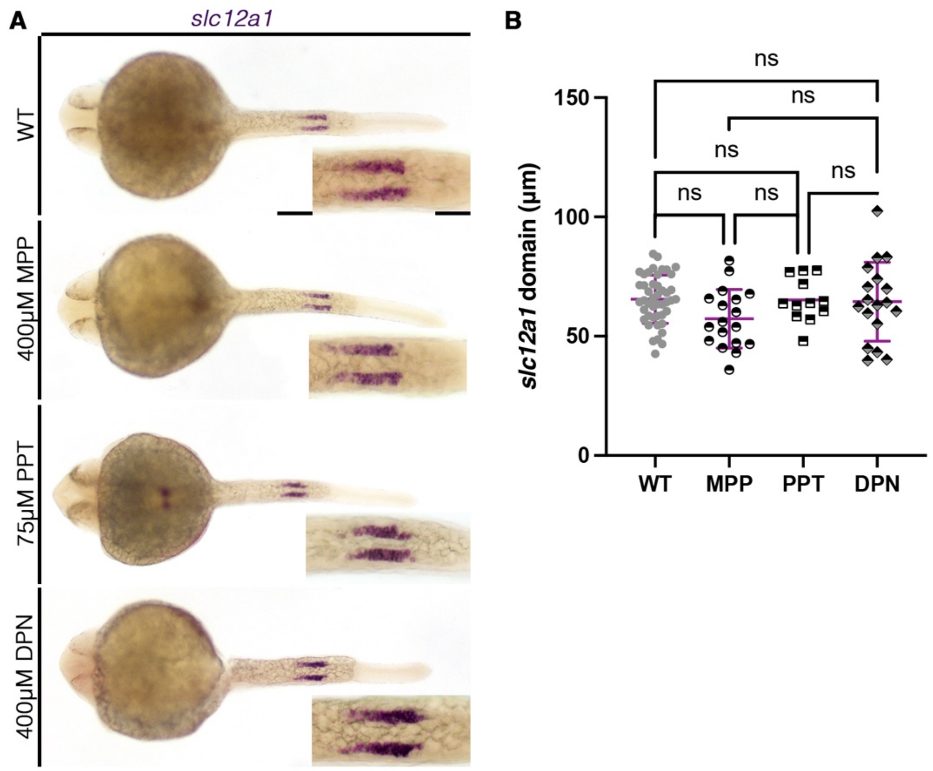

Supplemental Figure S5. Selective estrogen receptor modulators MPP, PPT and DPN have no effect on DE segment development in the zebrafish pronephros. (**A**) 28 ss WT (top), MPP (second row), PPT (third row) or DPN (bottom) treated animals stained via WISH for the DE marker (*slc12a1*). Scale bar = 100 µm for lower magnification, scale bar = 50 µm for higher magnification. (**B**) DE domain length at 28 ss in micrometers. Data presented on graphs are represented as mean ± SD; (ANOVA).

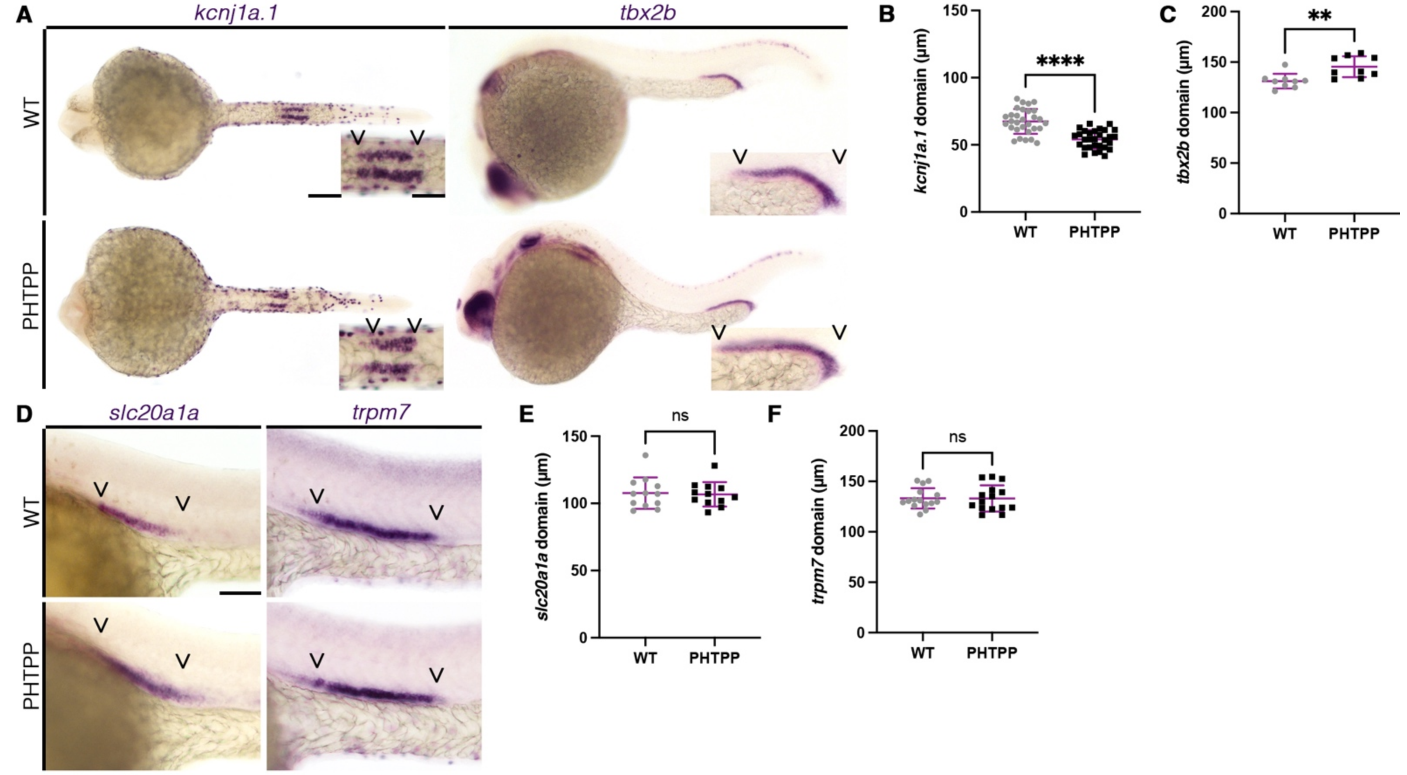

Supplemental Figure S6. Effects of Esr2 antagonist PHTPP on nephron segmentation. (**A**) 28 ss WT (top) and 18 µM PHTPP (bottom) treated animals stained via WISH for the DE marker (*kcnj1a.1*) (left), or DL marker (*tbx2b*) (right) Scale bar = 100 µm for lower magnification, scale bar = 50 µm for higher magnification. (**B,C**) DE, DL domain length at 28 ss in micrometers. (**D**) 28 ss WT (top) and 18 µM PHTPP (bottom) treated animals stained via WISH for the PCT marker (*slc20a1a*) (left) or PST marker (*trpm7*) (right). Scale bar = 50 µm. (**E,F**) PCT, PST domain length at 28 ss in micrometers. Data presented on graphs are represented as mean ± SD; **p< 0.01 and ****p < 0.0001 (t test or ANOVA).

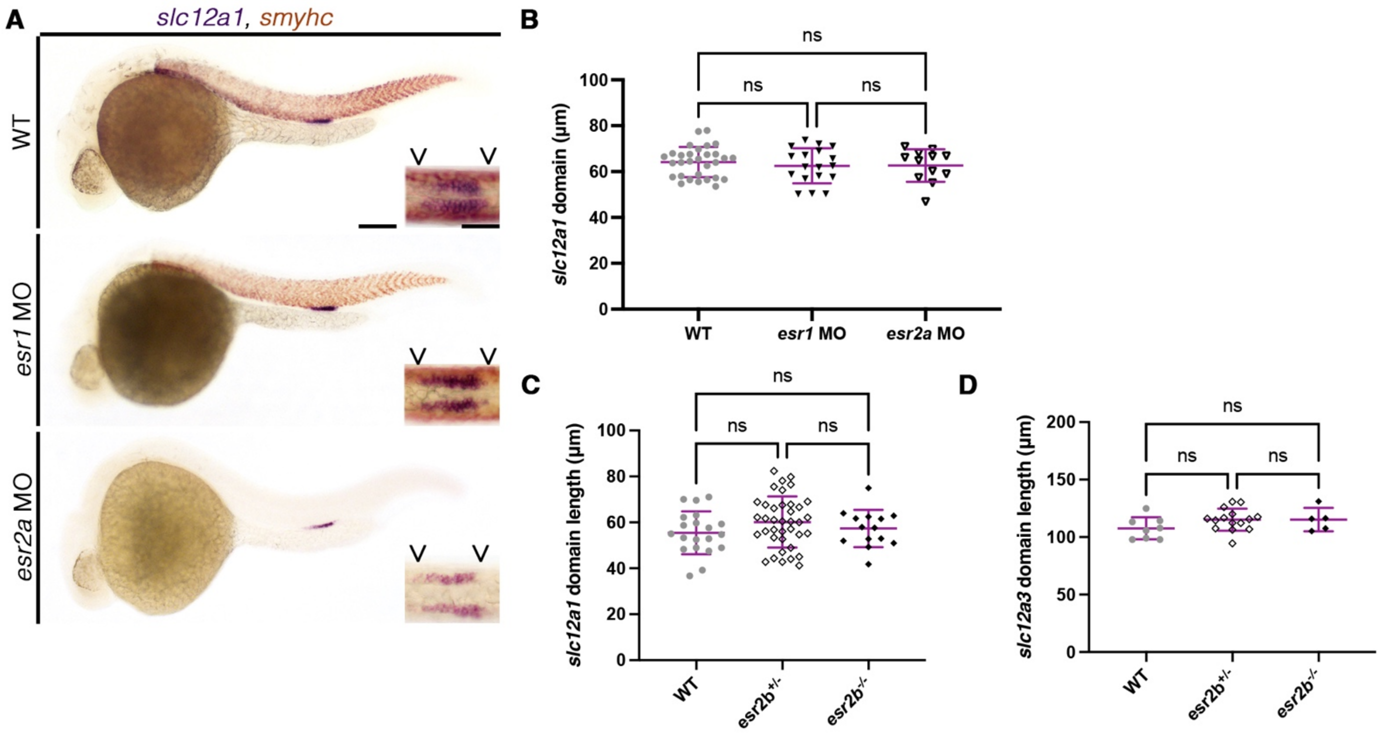

Supplemental Figure S7. Evaluation of DE segment development following *esr1* and *esr2* knockdown. (**A**) 28 ss WT (top), *esr1* MO (middle), or *esr2a* MO (bottom) injected animals stained via WISH for the DE marker (*slc12a1*) and somite marker (*smyhc*). Scale bar = 100 µm for lower magnification, scale bar = 50 µm for higher magnification. (**B**) DE domain length at 28 ss in micrometers. (**C**) DE domain length at 28 ss in micrometers of $esr2b^{-/-}$ and siblings. (**D**) DL domain length at 28 ss in micrometers of $esr2b^{-/-}$ and siblings. Data presented on graphs are represented as mean ± SD; (ANOVA).
